# Improved Method for *Ex Ovo*-Cultivation of Developing Chicken Embryos for Human Stem Cell Xenografts

**DOI:** 10.1155/2013/960958

**Published:** 2013-03-11

**Authors:** Timo Schomann, Firas Qunneis, Darius Widera, Christian Kaltschmidt, Barbara Kaltschmidt

**Affiliations:** ^1^Molecular Neurobiology, University of Bielefeld, Universitätsstraße 25, 33501 Bielefeld, Germany; ^2^Cell Biology, University of Bielefeld, Universitätsstraße 25, 33501 Bielefeld, Germany

## Abstract

The characterization of human stem cells for the usability in regenerative medicine is particularly based on investigations regarding their differentiation potential *in vivo*. In this regard, the chicken embryo model represents an ideal model organism. However, the access to the chicken embryo is only achievable by windowing the eggshell resulting in limited visibility and accessibility in subsequent experiments. On the contrary, *ex ovo*-culture systems avoid such negative side effects. Here, we present an improved *ex ovo*-cultivation method enabling the embryos to survive 13 days *in vitro*. Optimized cultivation of chicken embryos resulted in a normal development regarding their size and weight. Our *ex ovo*-approach closely resembles the development of chicken embryos *in ovo*, as demonstrated by properly developed nervous system, bones, and cartilage at expected time points. Finally, we investigated the usability of our method for trans-species transplantation of adult stem cells by injecting human neural crest-derived stem cells into late Hamburger and Hamilton stages (HH26–HH28/E5—E6) of *ex ovo*-incubated embryos. We demonstrated the integration of human cells allowing experimentally easy investigation of the differentiation potential in the proper developmental context. Taken together, this *ex ovo*-method supports the prolonged cultivation of properly developing chicken embryos enabling integration studies of xenografted mammalian stem cells at late developmental stages.

## 1. Introduction

The chicken is a well-studied and cost-efficient model organism profiting from a great potential of *in vivo* manipulation techniques. As early as the 5th century B.C. Hippocrates and later on in the 4th century B.C. Aristotle studied embryonic development using chicken embryos. More than 2000 years later, in 1951, Hamburger and Hamilton classified the developmental stages of the chicken embryo in 46 HH stages [1] allowing temporally defined manipulations in developing embryos. 

Using this kind of age-classification several *in ovo* experiments such as investigations on neural crest cells (NCCs) and their migratory behavior in the avian embryos were performed [2]. In this regard, stem cells obtained from different animals or even of human origin can be characterized for their potential neural crest ancestry. In a recent study, we transplanted human inferior turbinate stem cells (ITSCs) into early chicken embryos (HH15–HH18) [3]. The injected ITSCs migrated laterally forming chains, a characteristic hallmark of neural crest cells. In other studies by Soundararajan et al. and Son et al., motor neurons derived from embryonic stem cells as well as induced motor neurons reprogrammed from mouse and human fibroblasts were shown to integrate after transplantation into the chicken neural tube [4, 5].

For the investigation of developing chicken embryos, Auerbach and coworkers designed a method allowing long-term cultivation of chicken embryos in an *ex ovo*-setup [6]. In 1989, the containment for *ex ovo*-cultivation was improved concerning the short-term survival using a plastic cup covering the developing embryo with a petri dish [7]. This method permits easy access to the embryo as well as to the blood vessels of the chorioallantoic membrane (CAM). Besides the observation of the development, *ex ovo*-cultivated chicken embryos can be used for the investigation of toxicity of different substances in a vertebrate model. In this context, a shell-less cultivation method was used to observe the influence of nicotine and cigarette smoke in developing chicken embryos [8]. In addition, the effects of acute glucose toxicity could be assessed in shell-less chicken embryo cultures [9].

Recently, Yalcin and colleagues described an *ex ovo*-cultivation method of chicken embryos, which is suitable for microsurgical and imaging applications [10]. However, eggs were incubated for 72 hours previous to the transfer into the *ex ovo*-setup, and if cultivated beyond embryonic day (E) 7, crushed eggshell was added to achieve HH38, correlating with E12.

Here, we describe an inexpensive reusable shell-less cultivation method for chicken embryos in a broadly available containment. Using a defined amount of water and ground eggshell, we demonstrate for the first time the survival of *ex ovo*-cultivated embryos for at least 13 days up to E15 and HH stage 41. Moreover, in contrast to methods previously described, the herein presented method supports xenografts into late stages of *ex ovo*-cultivated chicken embryos.

## 2. Results

### 2.1. *Ex Ovo*-Cultivated Chicken Embryos Reveal Normal Morphological Development for 13 Days *In Vitro*

**Figure 1:**
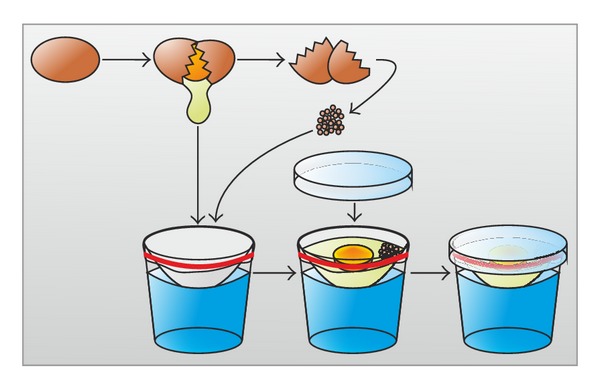
Schematic view of initial steps in *ex ovo*-cultivation of chicken embryos. Prior to transfer, chicken embryos were incubated at 37.8°C for 48 hours. Eggs were gently opened using a jigsaw. The indentation was expanded, and the contents were carefully transferred onto the support film. Ground eggshell of several eggs was added to the albumin and the experimental setup was covered using a bacterial dish.

**Figure 2:**
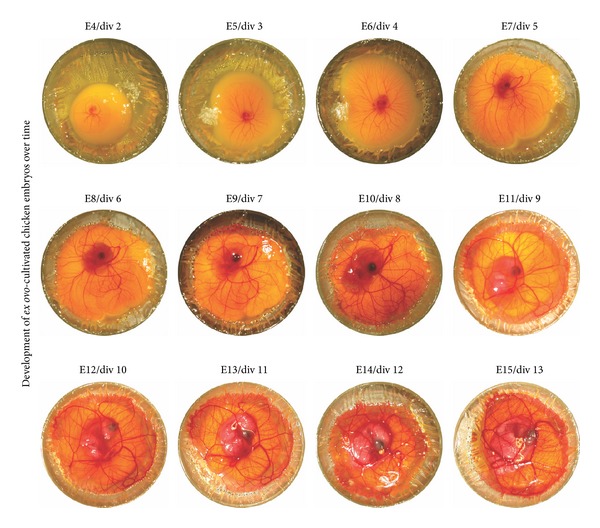
*Ex ovo*-cultivated chicken embryos show normal development over time. Starting with an easily visible chicken embryo at E4, morphological changes in the development of *ex ovo*-cultivated embryos are distinguishable. After the start of the *ex ovo*-cultivation, the embryo develops normally until E15 up to HH stage 41. The set of photos consist of a number of 4 different chicken embryos.

For the herein described *ex ovo*-cultivation method, egg contents of chicken eggs preincubated for 48 hours were gently transferred into a readily prepared shell-less containment, as demonstrated schematically in Figure 1. Development and morphology were compared to “normal stages of the chicken embryo” described by Hamburger and Hamilton [1]. As shown in Figure 2, embryos were easily detectable at E4 revealing normal development. During incubation, the yolk expanded on the support film of the *ex ovo*-containment, and the blood vessels started to span smoothly over the yolk. Furthermore, continuous observation showed normal morphological development of embryos up to E15, correlating with HH stage 41.

### 2.2. *Ex Ovo*-Cultivated Chicken Embryos Show Improved Survival without Significant Differences in Size and Weight

**Figure 3:**
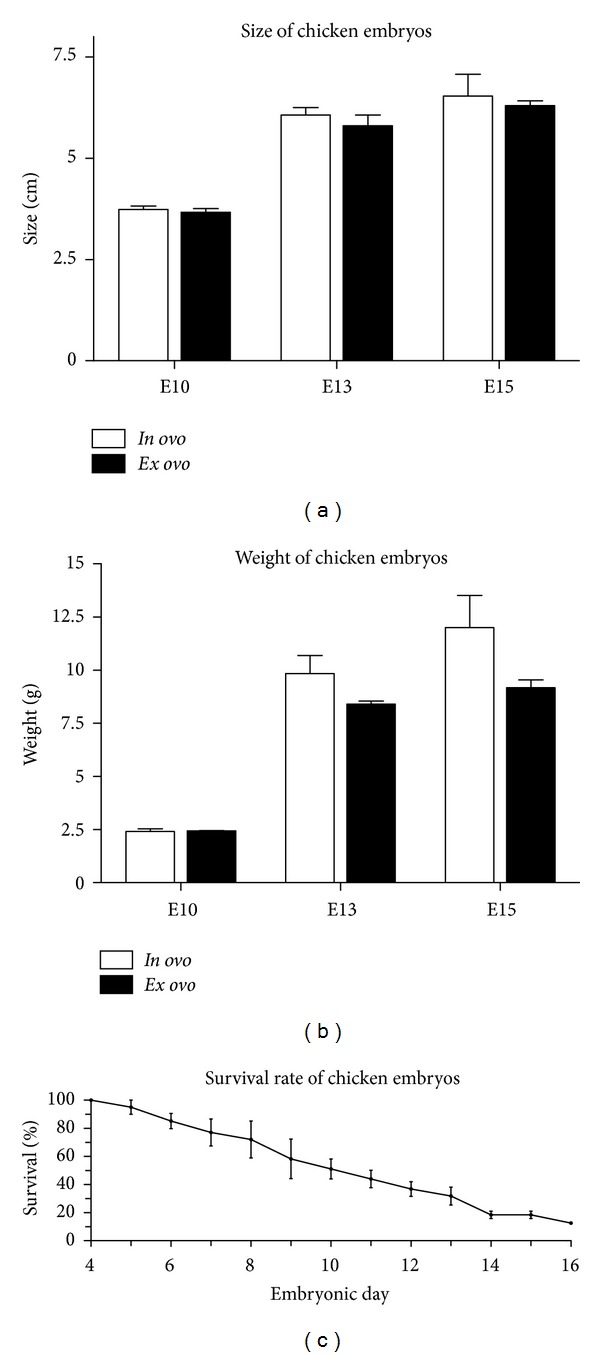
Statistical analysis of *ex ovo*-cultivated chicken embryos. (a) Comparison of size of *in ovo*- and *ex ovo*-cultivated chicken embryos on days E10, E13, and E15. No significant differences in size were detectable between shell-less and traditionally cultivated embryos. Error bar indicates standard error of the mean (SEM), *n* = 3. (b) *In ovo-* and *ex ovo-* cultivated chicken embryos were investigated regarding their size on days E10, E13, and E15 of incubation. Shell-less cultivated embryos revealed slightly decreased weight at E15 of incubation. Error bar indicates SEM, *n* = 3. (c) Survival rate of *ex ovo*-cultivated chicken embryos over time. After 48 hours of traditional incubation, egg contents were transferred into a shell-less containment and incubated for at least 13 days *in vitro*. Starting with easily visible embryos at E4 (4 independent cohorts, 11 embryos each), survival rate was determined for *ex ovo*-cultivated chicken embryos up to E16, by visual inspection of vital signs (heartbeat and movement) each day. Dead embryos were removed from the incubator. At E16 one living embryo was observed.

To investigate the size and weight, *in ovo*- and *ex ovo*-cultivated developing chicken embryos were sacrificed and compared at E10 as well as at E13 and E15 (Figure 3). Here, no significant differences in size were detectable between shell-less and traditionally cultivated chicken embryos. In addition, the embryos showed no significant differences in weight at E13 and E15 when incubated in a shell-less containment. We estimated the survival rate for embryos cultivated for 13 days *in vitro* (4 cohorts, 11 embryos each). Starting with easily visible chicken embryos, the survival rate was measured starting at E4 of *ex ovo*-cultivation. The addition of cell culture medium did not enhance the survival rate of chicken embryos (data not shown), which is contrary to the observations made by Auerbach and coworkers [6]. Importantly, cultivated in a humidified incubator at 37.8°C, more than 18% of the embryos were able to survive until E15.

### 2.3. Chondrogenesis, Osteogenesis, and Myelination of Nerves* Were Not Impaired by Ex Ovo*-Cultivation

**Figure 4:**
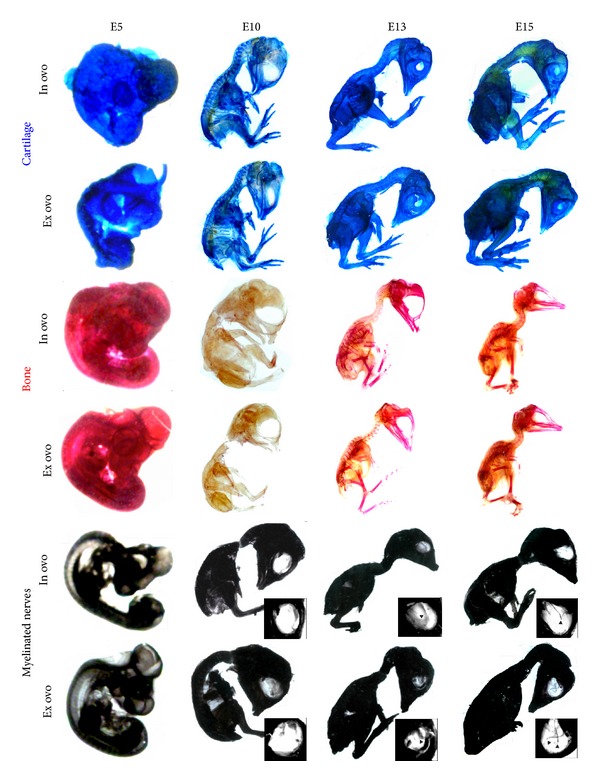
Histochemical stainings of cartilage, bone, and myelinated nerves of chicken embryos revealed normal chondrogenesis, osteogenesis, and myelination of the nerves. Chicken embryos cultivated either *in ovo *or *ex ovo *were sacrificed at E5, E10, E13, and E15 and subsequently fixed using 4% PFA. Afterwards, specimen was skinned and eviscerated followed by staining at 37°C overnight. Destaining in a graded series of ethanol was followed by clearing in 1 : 2 BABB/ethanol, 2 : 1 BABB/ethanol, and 100% BABB (1 : 2 benzyl alcohol/benzyl benzoate). Upper panel: 0.3% Alcian Blue staining solution in 70% ethanol and addition of 5% acetic acid for cartilage at E5, E10, E13, and E15 of *in ovo*- and *ex ovo*-cultivated chicken embryos. Middle panel: comparison of osteogenesis at E5, E10, E13, and E15 using 0.1% Alizarin Red S staining in 95% ethanol. Lower panel: staining of lipids using 5% saturated Sudan Black B in 70% ethanol indicates myelination of the optic nerves in E13 and E15. Arrowheads show the ending of optic nerves in the blowup.

Development of *in ovo*- and *ex ovo*-cultivated chicken embryos was compared at given time points regarding chondrogenesis, osteogenesis, and myelination of the optic nerves. At E5, the vertebrae of chicken embryos started to undergo chondrification [11]. In contrast to E5 embryos, which did not show specific staining for cartilage, E10 chicken embryos were positive for Alcian blue staining suggesting that cartilage and bone tissue of the chicken embryo started to chondrify at this point of time (Figure 4). At E13 and E15 of development, no differences in chondrification between *in ovo*- and *ex ovo*-cultivated chicken embryos were observed. Regarding osteogenesis, chicken embryos at E5 showed no specific staining as expected (Figure 4). At E10, the wings, skull, and ribs began to ossify. However, in the respective tissues no specific staining for bone was observed in any of the analyzed chicken embryos. In contrast, at E13, *ex ovo*- and *in ovo*-cultivated chicken embryos showed distinct staining for bone at comparable amounts. Up to E15, *ex ovo*-cultivated embryos showed normal osteogenesis in comparison to *in ovo*-cultivated chicken embryos.

We applied Sudan Black B to specifically stain lipid-rich myelinated nerves within *ex ovo*-cultivated chicken embryos [12–14]. Focusing on the myelination of the optic nerves the orbital cavity was investigated at E10, E13, and E15. Chicken embryos at E10 did not show specific staining for myelinated optic nerves. However, in E13, chicken embryos staining of the optic nerve could be observed (Figure 4). A more distinct staining of the optic nerve at E15 indicated advanced myelination.

### 2.4. Xenografted ITSCs Integrate in the Basal Layer of the Epidermis of *Ex Ovo*-Cultivated Chicken Embryos

**Figure 5:**
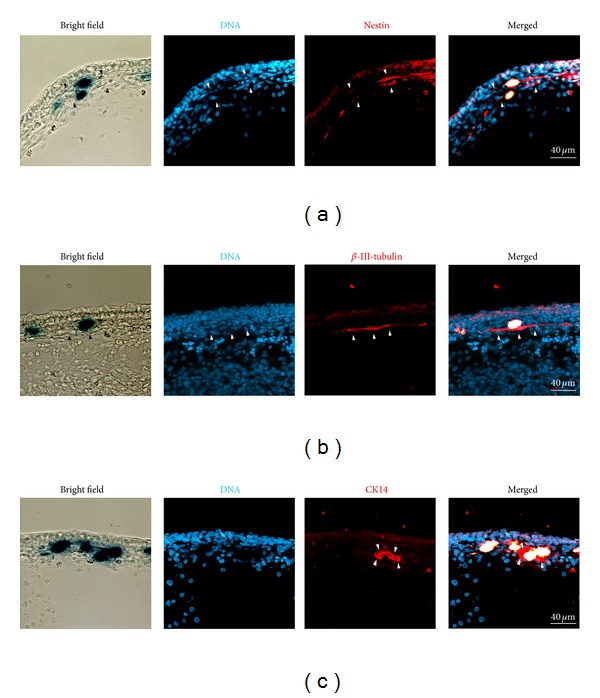
Immunohistochemical analysis of xenografted ITSCs. (a) Cryosections of embryonic chicken tissue harboring transplanted, human lacZ-positive cells. Xenografted integrated ITSCs revealed typical nestin expression (arrowheads). (b) Integrated ITSCs were positive for *β*-III-tubulin (arrowheads). (c) Integration of ITSCs in the basal layer of the epidermis was underlined by expression of CK14 (arrowheads) of xenografted cells. Transplantation experiments were performed as quadruplicate with consistent results. Representative results are shown.

For xenografts into developing chicken embryos, ITSCs were virally transduced using lentivirus harboring the lacZ-gene leading to a deep blue color of the cell nuclei after *β*-galactosidase staining. Using the here-described *ex ovo*-cultivation method, labeled ITSCs were injected into developing chicken embryos as late as HH stages 26 to 28, correlating with E5 to E6. An adequate time span of up to 4 days allowed proper integration and differentiation of xenografted adult human stem cells. Subsequently, manipulated chicken embryos were sacrificed followed by fixation and staining for lacZ-positive ITSCs. Tissue containing lacZ-positive stem cells was sectioned and stained using specific antibodies. LacZ-positive ITSCs injected into lesioned developing chicken embryos remained positive for the neural crest stem cell-marker nestin after 4 days, as demonstrated in Figure 5. Furthermore, xenografted ITSCs showed expression of the ectodermal marker *β*-III-tubulin suggesting partial phenotypic switch towards ectodermal lineage *in vivo*. Interestingly, transplanted ITSCs also showed expression of the basal cell-marker cytokeratin 14 (CK14) pointing towards a basal cell-like differentiation. This observation was underlined by bright field microscopy showing integration of ITSCs into the basal cell layer of the epidermis.

Taken together, virally transduced ITSCs expressing the lacZ-gene were able to integrate into late stages of the developing chicken embryos after xenografting in *ex ovo*-cultivated chicken embryos.

## 3. Discussion

The herein described *ex ovo*-cultivation system allows survival of chicken embryos for up to embryonic day 15 and microsurgical transplantation of human NCSCs into the developing embryo at late stages (E5-7). Cultivated chicken embryos showed normal development, as demonstrated by proper osteogenesis, chondrogenesis, and myelination of nerves, as well as no significant differences to the *in ovo*-approach regarding their size and weight. 

Its cost efficiency make the chicken embryo ideal for investigation and manipulation of development processes using variety of experimental methods. However, most of the cultivation methods deal only with early stages of development since *in ovo*-experiments of late developmental stages are restricted by the necessity of windowing the shell as well as by strong vascularization and presence of membranes [15].

To investigate late developmental stages of chicken embryos, the shell-less or *ex ovo*-cultivation was established and subsequently improved regarding the short-term survival of chicken embryos [6, 7]. In 1999, Brooks and coworkers studied angiogenesis in 10-days-old chicken embryos by using shell-less culture systems [16]. Moreover, shell-less chicken embryo cultures were used to investigate functional importance of N-cadherin in the developing chicken limb by application of monoclonal N-cadherin-specific antibodies [17].

Nevertheless, these studies described *ex ovo*-cultivation at early stages of developing chicken embryos, particularly, at age prior to or at day 3 of incubation. Hamamichi and Nishigori as well as Datar and Bhonde used late stages of *ex ovo*-cultivated chicken embryonic development to examine the influence of nicotine in E7 embryos and effects of acute glucose toxicity, respectively [8, 9]. Recently, Leong and coworkers applied a chorioallantoic membrane (CAM) assay to investigate cell migration and metastatic growth of cancer cells in *ex ovo*-cultivated chicken embryo systems [18]. However, none of the studies applied microsurgical applications to the chicken embryo itself. Although Dhole and colleagues already reported an injection method into the vitreous of the eye of late stage *ex ovo*-cultivated chicken, the authors did not investigate behavior and survival of injected cells [19]. Apart from this, the survival rate of chicken embryos was postulated to be over 50% after transfer, but no time-dependent statistics were made. More recently, Yalcin and colleagues presented an *ex ovo*-culture system allowing the cultivation of chicken embryos up to HH stage 38, correlating with E12 [10]. Despite using microsurgical methods, those were only applied to early stages of developing chicken embryos without statistically analyzing the survival.

Extending these promising findings, the herein described *ex ovo*-cultivation method allows cultivation of chicken embryos starting at 48 hours (E2) of incubation up to E15 correlating with HH stage 41 and beyond. Interestingly, the survival rate of chicken embryos decreases over time below 18.4% for E15 embryos indicating absence of important factors for embryonic development in *ex ovo*-cultures. Remarkably, only one embryo survived the *ex ovo*-incubation until E16 indicating that E15 represents the latest possible time point of investigation. 

We further describe for the first time the utilization of microsurgical applications for experiments on late stages of *ex ovo*-cultivated chicken embryos at E5 and later. In this context, lacZ-positive ITSCs were stained for the ITSC-marker nestin after transplantation and integration into developing chicken embryos. This observation is consistent with expression in their endogenous niche of the human inferior turbinate [3, 20].

Although early stages of developing chicken embryos are used for the characterization of stem cells such as in case of chain migration of NCSCs, differentiated tissues of chicken embryos cultivated for a prolonged time more closely resemble late stages of development of an organism as in case of differentiated tissue of limbs. Therefore, information about the differentiation potential in already developed tissue such as bones, cartilage, the nervous system, or skin can be obtained.

Taken together, we describe an *ex ovo*-cultivation method suitable for long-term cultivation and investigation of chicken embryos. In a developmental context, normal growth was shown with regard to weight and size as well as cartilage, bone, and myelinated nerves of embryos. Moreover, the injection of human neural crest-derived ITSCs was performed to investigate the usability of the herein-described *ex ovo*-method with respect to microsurgical applications. Here, our method allows the use of late developmental stages of *ex ovo*-cultivated chicken embryos for microsurgical transplantation of stem cell xenografts. 

## 4. Experimental Procedures

### 4.1. Materials

- Reagents:
  (1)  distilled water,
  (2)  70% ethanol,
  (3)  ground eggshell.
- Chicken eggs
   fertilized nonincubated chicken eggs were obtained from a local  supplier (Brüterei “Brormann”, Rheda-Wiedenbrück, Germany).

#### 4.1.1. Equipment

- *Ex ovo*-cultivation:
  (1) glasses (GODIS, Art-nr: 800.921.09, IKEA, 7 3/4 oz. 23 cl),
  (2)  cling film,
  (3)  elastic bands,
  (4)  scalpel or razor blade,
  (5)  sterile bacterial petri dishes,
  (6)  incubator.

### 4.2. Methods

#### 4.2.1. Precultivation-Steps

- Incubate fertilized chicken eggs for 48 h at 37.8°C prior to *ex ovo*-cultivation.  *Caution:* see Note 1.
- Warm a humidified incubator to 37.8°C. *Tip:* see Note 2.
- Use ground eggshell as a source of calcium nutrition for the chicken embryo to  efficiently prolong the survival of embryos. *Caution:* see Note 3 *tip:* see Note 4.
- Prepare surrogate shell as follows:
  (1)  Fill glasses with 160 mL (~74% of total volume) distilled water. *Tip:* see  Note 5 *critical:* see Note 6.
  (2)  Preparation of support film: place a quadratic piece of cling film on top  of the glass. Carefully lower the film manually until an area of 4 cm-5 cm  in diameter is in contact with the surface of the water. *Caution:* see  Note 7.
  (3)  Fix the support film with an elastic band on the glass.
  (4)  Cut off excess cling film with a scalpel or razor blade.
  (5)  Place one side of a sterile bacterial petri dish as a lid on top of the glass.  *Critical:* see Note 8.
  (6)  Add up to 5 mL cell culture medium such as DMEM high glucose with  and without supplementation with penicillin and streptomycin (P/S) (5 mL/50 mg; PAA, Pasching, Austria), amphotericin B (amphoB) (5 mL/1.25 mg; PAA), L-glutamin (L-glu) (200 mM; Sigma-Aldrich),  and 10% of fetal calf serum (FCS). *Caution:* see Note 9.

#### 4.2.2. Transfer of Egg Contents and *Ex Ovo*-Culture

- Sterilize eggshells with 70% ethanol and wipe with a paper towel.
- Gently open eggs incubated for 48 h at 37.8°C laterally using a jigsaw (Figure 1). *Caution:* see Note 10.
- Saw until a dent puncturing the eggshell appears. *Tip:* see Note 11.
- Widen the dent this way up to 5 cm-6 cm laterally. *Critical:* see Note 12.
- Place thumbs besides the dent and turn the egg dent-side down. Gently pull the two pieces of eggshell apart at the dent. *Critical:* see Note 13.
- Let the egg contents gently flow onto the support film. *Tip:* see Note 14.
- Carefully add about 1 g ground eggshell to besides the embryo. *Caution:* see Note 15
- Cultivate chicken embryos within the surrogate shell and the bacterial dish on top at 37.8°C in a humidified incubator.

### 4.3. Notes

**Note 1**

Set a humidified incubator at temperatures between 37°C and 38°C; incubation of chicken eggs should not exceed 48 hours.

**Note 2**

Use autoclaved water containing 1 mM CuSO_4_ to prevent contamination.

**Note 3**

Sterilize the exterior of the eggshell with 70% ethanol and grind eggshell pieces to a fine powder.

**Note 4**

Prepare sufficient amounts of shell from several eggs at once and store remaining ground eggshell at −20°C for further setups.

**Note 5**

Autoclave glasses prior to use to prevent contamination.

**Note 6**

Use a defined amount of water; too much water may result in leakage of albumin; an insufficient amount of water may result in drop-related damage of the yolk as well as the embryo.

**Note 7**

Use sterile gloves to prevent contamination.

**Note 8**

Spray the support film with 70% ethanol for sterilization before placing the bacterial petri dish on top. Allow the ethanol to evaporate or remove it manually with a sterile paper towel prior to transfer of the egg contents.

**Note 9**

Addition of amphoB to the medium might result in decreased neurogenesis of chicken embryos.

**Note 10**

Sterilize jigsaw prior to use with 70% ethanol.

**Note 11**

Do not exert too much pressure on the shell. Simply slide the jigsaw back and forth until a dent appears.

**Note 12**

Avoid the leaking of egg white.

**Note 13**

While gently pulling the eggshells apart, hold the egg closely over the support film to avoid damage to the yolk and embryo.

**Note 14**

If the embryo is not located on top of the yolk, it will move there autonomously within 24 hours.

**Note 15**

Do not drop ground eggshell directly on the chicken embryo.

### 4.4. Variations

To achieve a prolonged survival Auerbach additives may be applied as follows:

- add 5–10 mL tissue culture medium to the surrogate shell,
- add 100–200 units/mL of gentamicin and mycostatin to the medium,
- incubate chicken embryos in an incubator with 1%-2% CO_2_.

#### 4.4.1. Addition of Ground Eggshell at E10

 At E10, the yolk and blood vessels are fully spread in the surrogate shell/shell-less containment, though addition of ground eggshell on the CAM might provide better accessibility to the supplement.
